# Combining PPI with qualitative research to engage ‘harder-to-reach’ populations: service user groups as co-applicants on a platform study for a trial 

**DOI:** 10.1186/s40900-016-0023-1

**Published:** 2016-03-24

**Authors:** Heather Morgan, Gill Thomson, Nicola Crossland, Fiona Dykes, Pat Hoddinott, on behalf of the ‘BIBS’ study team

**Affiliations:** 1 Health Services Research Unit, University of Aberdeen, Foresterhill, Aberdeen, AB25 2ZD Scotland, UK; 2 Maternal and Infant Nutrition & Nurture Unit (MAINN), School of Health, University of Central Lancashire, Preston, PR1 2HE England, UK; 3 Nursing, Midwifery and Allied Health Professions Research Unit, University of Stirling, Stirling, FK9 4LA Scotland, UK

**Keywords:** Public involvement, Qualitative research, Complex interventions, ‘harder-to-reach’ perspectives, Feasibility studies, Trial design, Service user collaboration, Participatory methods

## Abstract

**Plain english summary:**

It is recommended that research studies are carried out with or by patients and the public through their involvement from the beginning and in as many stages as possible (known as PPI). Some studies formally invite patients and the public to participate in interviews and focused group discussions to collect views about topics (known as qualitative research). In our study on financial incentives for giving up smoking in pregnancy and breastfeeding, we combined both PPI and qualitative research to include the views of women with a range of experiences of smoking and breastfeeding.

We involved two mother and baby groups in disadvantaged areas of North East Scotland and North West England as research partners on our team. First, we asked members to comment on our research plans and documents, which is standard PPI. Second, we asked members to participate in voice recorded discussions, contributing to qualitative research data. These discussions revealed different views from those that we heard through research interviews. They allowed us to develop more relevant research tools and resources. Members also helped us to identify people outside the groups who we could interview.

Combining involvement and participation helped us to include the views of a wide range of women from ‘harder-to-reach’ groups who don’t usually take part in research. This was important because the research was intended for women who could benefit from incentives to stop smoking in pregnancy and breastfeed, often present in such groups. Positive continuing relationships and trust improved on involvement or participation alone.

**Abstract:**

ᅟ

**Background:**

Patient and public involvement (PPI) in all research studies is recommended from the earliest point and in as many stages as possible. Qualitative research is also recommended in the early stages of designing complex intervention trials. Combining both together might enable inclusion of ‘harder-to-reach’ perspectives from the target population(s), particularly when the research is intended for their benefit. However, the interface between PPI and qualitative research has received little attention.

In a multi-disciplinary, mixed methods study to inform the design of incentive trials for smoking cessation in pregnancy and breastfeeding, we combined PPI and qualitative research, with some overlap. Mother and baby groups from two geographically separate disadvantaged areas, with diverse experiences of the smoking and breastfeeding, but no training or previous involvement in research, were recruited as PPI research grant co-applicants. An iterative partnership approach facilitated involvement in research conduct and design across all project phases. Group PPI members were also invited to contribute to more formal qualitative data collection, as and when indicated by the research questions, and emerging analysis.

**Results:**

We engaged with ‘harder-to-reach’ women in mother and baby group settings, rather than in academic or home environments. These settings were relaxed and informal, which facilitated rapport-building, disclosures of unexpected information and maintained trust. Twenty-one women participated in standard PPI activities: feedback on study protocols and documents; piloting questionnaires and interview schedules. PPI members voiced some different perspectives from those captured within the qualitative dataset. Nineteen participated in focused qualitative research. Novel aspects were audio recorded PPI discussions, which contributed qualitative data; first, to interpret systematic review findings and construct intervention vignettes for use in the qualitative research; second, to assist with recruitment to improve sample diversity in the formal qualitative dataset; and third, to translate theory and findings presented in a researcher generated logic model into a lay tool. This had face validity for potential trial participants and used the metaphor of a ladder.

**Conclusions:**

Combining and overlapping PPI and qualitative research added ‘harder-to-reach’ contributions, sample diversity, trust and engagement in creative approaches beyond what could be achieved through PPI or qualitative research alone.

**Electronic supplementary material:**

The online version of this article (doi:10.1186/s40900-016-0023-1) contains supplementary material, which is available to authorized users.

## Background

Patient and public involvement (PPI) in research entails working with patients (people with a medical condition receiving health service treatment), public (residents of the country) and people who use services. The purpose is to enhance depth, credibility and applicability of findings, improve clarity of research reports and recommendations and ensure immediate links between practice-based evidence and evidence-based methodology [1–3]. It is recommended in medical and health services research, including clinical trials, especially when funding is through taxation. The importance of incorporating PPI into research is acknowledged internationally, e.g. Ottawa Charter for Health Promotion 1986 (World Health Organization) [4], and nationally, e.g. US National Institutes of Health [5], and is enshrined in UK statutes, for example the *Health and Social Care Act 2001*, within the *Local Government and Public Involvement in Health Act 2007*, and more recent reforms contained within the *Health and Social Care Act 2012*, as well as in National Institute for Health Research [6] and other guidance [7, 8]. Qualitative interviews, focus groups and observations are also recommended in The Medical Research Council’s guidance on the development and feasibility testing of complex interventions to incorporate wider views and perspectives into the study direction [9].

Both PPI and qualitative research [9] should begin at the earliest stage possible for maximum benefit to all stakeholders [10–12]. Qualitative research has rigorous quality appraisal standards [13] and reporting guidelines, with particular attention paid to the rationale for the sampling strategy and reporting the sample characteristics [14]. While the descriptions of how PPI representatives are selected and their characteristics vary across studies, the recent GRIPP checklist [15] has outlined key issues that should be reported. However, there are still specific challenges when selecting PPI and qualitative research participants for prevention research topics where health inequalities are observed, for example lifestyle behaviours in pregnancy [16]. The most disadvantaged or marginalised in society, who are often the main target population for such interventions, are the hardest to access and engage [17]. ‘Harder-to-reach’ is often used to refer to individuals who have a low socioeconomic status (SES), are members of ethnic minorities, and who have a low level of literacy. However, it is a contested term as it can be perceived as pejorative and is usually underpinned by a number of preconceptions [18]. There are numerous barriers that can prevent people who are labelled as ‘hard-to-reach’ from being involved in research, e.g. personal characteristics, where people live, communication issues [19] Therefore, the ways in which research teams approach PPI vary [20]. In practice, early PPI with representatives of ‘harder-to-reach’ populations is not always easily achievable unless a formal, regular PPI arrangement already exists [21]. Advertising for volunteers may not attract typical members of the target population(s) [22]. Constraints on time and funding to proactively recruit appropriate PPI members can act as barriers, which can marginalise involvement in the crucial stages, i.e. the development of a research bid [22–25]. PPI rationale and selection procedures have historically lacked clear description. The GRIPP checklist has helped to address some of the key issues by highlighting what needs to be addressed: limited conceptualisation of PPI; poor quality of methods reporting; poor reporting of context and process; wide variability in the way impact is reported; little formal evaluation of the quality of involvement; limited focus on negative impacts; and little robust measurement of impact [15]. GRIPP 2 will soon be published. However, it can still be difficult to assess whether and how the PPI integrates perspectives from members of the intended target population(s) and into what aspects or stages of the research [6]. As a result, formal qualitative research tends to take precedence because methodologies and sampling are more systematically documented. PPI and qualitative research components are not proxies for one another and perform different, although often complementary, functions. They can, however, overlap and be combined, which might offer potential for including ‘harder-to-reach’ perspectives, though the interface between them has received little attention.

‘BIBS: Benefits of Incentives for Breastfeeding and Smoking cessation in pregnancy: a platform study for a trial’ [26] was multi-disciplinary and used mixed methods: we used both PPI and qualitative approaches, sometimes overlapping, as well as quantitative approaches. A platform study is one that considers the range of quantitative and qualitative evidence available around populations, interventions, comparators and outcomes to inform the design of a clinical trial. BIBS therefore comprised evidence syntheses, primary qualitative research and surveys (including a Discrete Choice Experiment (DCE), which is a quantitative technique for eliciting preferences that can be used in the absence of revealed preference data. The method involves asking individuals to state their preference for hypothetical alternative scenarios, goods or services). We also aimed to understand and incorporate target population perspectives into all stages through PPI *and* by including representatives’ participation in the formal qualitative research component. We set out to foster a partnership approach [27], emphasising interactive communication, with what are locally considered by health and social care services to be ‘harder-to-reach’ women who are more likely to smoke in pregnancy and not breastfeed, and who are targeted for participation in community groups. These groups aim to engage mothers living in less privileged postcode areas who are encouraged by health or community staff to attend for health and social care support. While women were not specifically ‘recruited’ to attend, the outreach workers at the Children’s Centre and health-care staff (i.e. midwives, health visitors) actively encourage all mothers in the local community to access the group for social and emotional support. We worked with both groups as co-applicants early on in order to guarantee inclusion and engagement of populations with whom the target behaviours of smoking in pregnancy and not breastfeeding are typically associated [16, 26]. (See also Appendix 1) Service user involvement was therefore conceived as an iterative, loosely structured process that was emergent and sensitive to their views and preferences in respect of our research conduct, whilst also enabling us to access and include ‘harder-to-reach’ perspectives through formal qualitative research activities, including recruitment to the main sample. The aim of this paper is to describe and reflect on the PPI and its interaction with the formal qualitative research components undertaken in our study.

## Methods

Our multi-disciplinary, mixed methods platform study to inform the design of incentive trials for smoking cessation in pregnancy and breastfeeding was commissioned by a UK government funding body that involves service user representatives in decision-making about research priorities [28]. In the UK, these commissioned calls have tight turnarounds, with the deadline for outline application 8–12 weeks after advertisement, and a further 8 weeks for a full application if shortlisted. No pre-existing PPI arrangements were suitable for this study where the target population for financial incentives would be women who smoke in pregnancy and who do not breastfeed. As such, these women are a ‘harder-to-reach’ group and we adopted an innovative approach by actively engaging service users from two mother and baby groups as co-applicants. These groups were selected because they were located in the most disadvantaged postcode areas of the cities closest to the academic centres leading the PPI and qualitative research in the BIBS project. This helped to facilitate involvement as the researchers had fairly close proximity with the groups and hence could be more flexible with attending meetings.

### Identification of service user groups

At the grant application stage, we approached maternity services, primary care and children’s centre managers to ask them to suggest two thriving, but diverse, mother and baby groups operating in areas of high deprivation in North East Scotland and North West England where smoking in pregnancy and formula milk feeding are prevalent. Such women infrequently attend health promoting services [16], and do not tend to participate in PPI and research initiatives; thus their perspectives are less often heard. [17] Smoking in pregnancy and not breastfeeding are also more likely for younger, less educated women living in more disadvantaged areas [16], and therefore involving perspectives from these populations, included within the memberships of the groups, was all the more important. Managers identified group liaison staff to negotiate and represent group involvement in the pre-funding study discussions: a health visitor (Group 1) and a mother and baby group coordinator (Group 2). The groups themselves were named as the co-applicants. The groups differed: Group 1 was user-led, successful in generating sustainability funding and provided a café and crèche as incentives to attend. Group 2 was based in a Local Authority Children’s Centre, where a previous peer support service delivering incentives to improve breastfeeding outcomes had been located [29].

### Approach

The respective group representatives were sent drafts of the grant proposal to discuss with the groups, and group members provided feedback via the representatives during the research planning stages in 2010. They negotiated reimbursement for their group’s refreshments and/or crèche costs as part of the grant application (£960.00 each, spread over three or four payments spanning 20 months). Although the study was on incentives, this was not offered as a financial incentive for participation, but was reimbursement for use of premises, refreshments and crèche provision as a research cost.

In the UK, National Health Service (NHS) ethics committee approval is required for recruitment of qualitative research participants through health services, and research sponsors have ethics committees and procedures for research recruitment in the community. Our service users were recruited as co-applicant groups by health and social care professionals who co-opted them as members of the research team at the application preparation stage. PPI does not usually require ethics approval [30], although there are principles and indicators of successful PPI in NHS research that raise ethical issues [31, 32]. Our approach meets the thirteen principles outlined for good PPI in NHS research (see Table 1) [31, 32].

**Table 1:** Principles of successful consumer involvement in NHS research

1.	The research will lead to benefits for consumers, in terms identified by the consumers themselves;
2.	Consumers are involved in every stage of the research, from identifying the research area through to sharing the research findings;
3.	Consumers’ expectations of being involved in the research are made clear to the researcher;
4.	The roles of consumers are agreed between the researchers and consumers involved in the research;
5.	Consumers have the opportunity to engage in research in the manner and at the level they wish, opting out of being involved in research at any time;
6.	Researchers budget appropriately for the costs of consumer involvement in research;
7.	Consumers are from sections of society and walks of life that are appropriate to the research;
8.	Researchers respect the differing skills, knowledge and experience of consumers;
9.	Consumers are offered training and personal support, to enable them to be involved in research;
10.	Researchers ensure that they have the necessary skills to involve consumers in the research process;
11.	Consumers are involved in decisions about how participants are both recruited and kept informed about the progress of the research;
12.	Consumer involvement is described in research reports;
13.	Research findings are available to consumers, in formats and in language they can easily understand

Our participatory research approach was at the interface between PPI and qualitative research and the principles do not account for ethical approvals which might be required where there is such overlap. Therefore, we considered it necessary to obtain ethics approval to gain each woman’s informed consent to actively participate in the study and to be able to use verbatim quotations in study publications. Because the groups had not been involved in research before, we wanted to engage on women’s own ‘turf and terms’, in addition to inviting women to project meetings at the University. From the start, we anticipated that we might want to audio record some of our discussions to include as more formal qualitative research. Recording and the potential to use quotes as data in reports distinguishes PPI from qualitative research, as any recording is potentially identifiable and rigorous data management procedures are required under the *Data Protection Act 1998*. We also wanted to collect socio-demographic data, where possible, and a history of participants’ infant feeding and smoking behaviours to ensure broad representation of the target population. This creates tensions around the optimum timing of ethics applications at the interface between PPI and qualitative research. We wanted external ethical review to ensure that our approach was consistent with the Helsinki declaration [33] and not over-burdening service users. When the study started (February 2012), service users assisted in developing the protocol and study information materials through third-party feedback to the group representatives whilst we applied for ethical approval. Approval was granted by the National Research Ethics Service, North of Scotland, on 10 May 2012 (*Research Ethics Committee reference: 12/NS/0041; Protocol number: 2/024/12* [34]*).* Once ethical approval had been obtained, the group representatives introduced the researchers (HM to Group 1 and GT to Group 2) to the service users. The two researchers led the PPI and the formal qualitative recruitment, data collection and analysis for the study.

### Group characteristics

A summary of the group characteristics is presented in Table 2 and described in depth below.

**Table 2:** Co-applicant mother and baby group profiles

Group	Location; distance from University	Setting	Average attendance	Meetings	Purpose and structure	Funder	Group meetings attended by researcher	Total individual participants formally engaged	Meetings contributing to formal qualitative data collection (audio recorded)
Group 1	North East Scotland; ~10 minute walk	One room within a larger family centre, including a separate crèche room. Sofas, play area adjacent, toys, kitchen area, dining area, unobtrusive site manager in room nearby	~7 mothers (2 mothers left the group and 2 new mothers joined) and their babies, 2 grandmothers – fairly regular group membership	Weekly, Wednesdays noon-2 pm (except during school holidays), plus some additional social/fundraising events	Set format: the café is facilitated by mothers who make and sell a cheap, healthy lunch costing £2 (subsidised through fundraising) to mothers and babies during the first hour. During the second hour, the babies go into the crèche and the mothers enjoy a coffee and a catch up, or participate in training activities/external speakers	Established through a partnership project between Aberdeen City Council, *Homestart* (http://www.home-start.org.uk/) and NHS Grampian. The café crèche is now independent and self-funding	*n* = 11 (+ *n* = 3 social events)	*n* = 9	Two focus groups (*n* = 5; *n* = 5); logic model session (*n* = 4)
Group 2	North West England; 18 mile drive	One room within a larger community centre, with toys, books and soft play facilities and seating for parents. A café is also located in this room for parents to purchase drinks and food	16-20 families (discontinuous participation)	Weekly, Fridays 9 am-noon (except during school holidays)	Unstructured format. Mother and baby/toddler group in an informal setting where parents can drop in and out to interact with and receive support from Children’s Centre staff members and engage with peers. Children Centre staff member in attendance throughout the session	Local government	*n* = 4	*n* = 12	One focus group (*n* = 4); individual interviews (*n* = 2); logic model session (*n* = 4)

### Settings and involvement

An unusual aspect of our study is the involvement of the research team with established community groups in their own environments during their usual meeting times. The research was not an ‘add on’ and no additional time commitment was requested. Negotiating this engagement was crucial to the success of the involvement we achieved from ‘harder-to-reach’ women, particularly as these women were caring for babies and older children.

At both sites, attendance at group meetings initially consisted of building a rapport with the members and observing in an unobtrusive manner to acknowledge the researchers’ roles as visitors to established groups [35]. Participatory approaches were adopted for PPI to facilitate group-directed involvement [36]. We wanted the groups to guide the development of our research partnerships in their usual group meetings, but to have flexibility to address specific research questions through audio recorded group discussions which would contribute qualitative data to the study. We worked to their timetables regarding convenient visiting times to either involve members or to formally collect data. This resulted in different patterns of engagement at each site.

#### Group 1

Contacts were made through the health visitor group representative to begin with, the first visit having been advertised in advance with a flyer and through circulation of reminder information leaflets. However, the researcher (HM) was able to exchange contact details with group members at her second visit at which point the gatekeeper role shifted from the health visitor to the group’s treasurer (a mother) and secretary (a grandmother). They suggested appropriate weeks to attend for participatory visits. Informed written consent to contribute to research activities was gained from each member at the first visit and from new members on subsequent visits. Women were also requested to complete socio-demographic, smoking and breastfeeding experience characteristics forms. (See Additional file 1).

Communicating directly with members of Group 1 by email and Short Message Service (SMS) allowed for less formal communication, good rapport-building and thus intermittently ‘dropping in’ for lunch visits, as well as the more directed research sessions, eased by the close proximity of the group to the University (~10 min walk). In February/March 2013, the health visitor support for the group ceased following a change of personnel and service reorganisation; however, this did not adversely affect engaging service users and possibly strengthened a direct partnership between the researcher and the group. Health professional/community staff presence was observed as inhibiting the spontaneity of contributions. Group 1 used *Facebook* (www.facebook.com) as its primary communication mode; however, despite gaining ethical approval to do so, the research team decided against trying to engage with the group using this social media. Several concerns were discussed around maintaining confidentiality and objectivity for the researcher during the study, appropriate boundaries, best use of researcher time and a lack of organizational guidance on research use of social media. Using email/phone/SMS only, however, did not appear to prevent or diminish involvement.

To begin with, the researcher stood passively on the periphery of the meeting room, waiting to be invited in. Eventually, and over the course of the collaboration, she was encouraged to join women sitting comfortably on the sofas, or on the floor. She also more actively engaged when she was asked to console crying babies, make tea and wash dishes. During the meetings, women sat around a large coffee table or leaned in over the kitchen counter if they were making the tea/coffee. Meanwhile, their children were occupied in the crèche, which meant that the researcher could interact with women both more and less formally.

The group environment, cohesiveness and set structure at Group 1 suited focused activities to address research questions and collect data. Women in this group were familiar with engaging in courses (e.g. food preparation, child first aid) and had regularly welcomed external speakers in the second hour of their weekly two-hour meetings. Computing/projector facilities were not available, so a white board and printed interactive materials were spread out on a low table for developing vignettes and testing a ladder logic model both discussed below [37]. A logic model is a tool used by funders, managers, and evaluators of programs to help understand how a program is likely to work). These uninterrupted, audio-recorded and focused activity discussions enabled service users to be positively engaged in the study in a more concrete and tangible way. However, some voices were dominant and there was a hierarchical structure and group membership rules that had seemed to have emerged over time. On one occasion, internal politics caused tension and prevented research activities taking place because members disputed rota duties (e.g. washing up and cleaning the carpets). This shows that the presence of the researcher did not affect normal group dynamics, which may have been the case had involvement taken place within a University setting or if a formal focus group had been arranged as is usual in qualitative research. Regularly taking the research to the groups, and thus reaching out to people in their communities to make involvement easier [19], enabled ‘harder-to-reach’ and more confident PPI than members of the research team have experienced when group representatives have been invited to the university setting. This methodological strategy avoids the selection bias that can occur if group members are asked to volunteer to participate in additional research activities outside usual group time. Previous experience suggests that only the more confident individuals accept such invitations and their views may differ from those who decline such invitations. Women involved in PPI could access support within their groups from the attached health visitors and/or social care professionals. Our approach contrasts with more ‘professionalised’ PPI, where cumulative experience and training can limit PPI representatives’ ability to offer a patient or lay perspective [38]. Although there can be benefits to involving trained PPI representatives [6], it was important that representatives of our study’s target population maintained their own perspectives, rather than by training them in contributing PPI to research from academic, health services research or clinical perspectives We wanted as far as possible to seek maximum diversity and disconfirming perspectives which is appropriate in the early stages of trial design. Our focused activity discussions emerged as a strategy to meld PPI with qualitative research and thus to broaden our sample.

#### Group 2

More formal meetings emerged as the most appropriate strategy for engagement with Group 2. Whilst an outline of the study had been provided to the group by the coordinator prior to the initial visit, the researcher (GT) initiated all direct contact with the women. The distance of travel to the group (18 miles by car) was a barrier to informal contacts and attendance was negotiated through the mother and baby group coordinator. When the researcher wanted to attend, email communication occurred at least two weeks in advance. During each visit, the researcher circulated and chatted to mothers informally prior to discussing the research. The use of a large room within the Children’s Centre meant that the researcher took a more opportunistic approach to involving group members spread across the space. Informed written consent to contribute to research activities was gained from each member of the group. The group’s drop-in format presented the researcher with the practical challenge of obtaining informed written consent on an ongoing basis, or re-establishing it for those who had consented previously, together with completing the characteristics forms. (See Additional file 1).

### Standard PPI contributions

This involved contributions to decision-making about all stages of the research, including the design of information materials and what data should be collected, from which participants, and how. Group members also helped to pilot interview topic guides for the formal qualitative research, survey questionnaires and a DCE; collaborated in writing the lay summary; and dissemination of key findings of the study. These more standard contributions made by PPI to our study are detailed in Appendix 2 with the qualitative research being detailed in Appendix 3.

### Qualitative data collection combined with PPI

After establishing PPI relationships and gauging an appropriate level of involvement with our work through negotiation, we gradually introduced specific research questions into more focused activity discussions with the groups. This melded PPI with qualitative research in an iterative manner and sessions were audio recorded with participants’ informed consent [34]. We negotiated with group members that transcriptions of our audio recordings of group discussions would be included in formal qualitative data analysis and members were keen to have their views incorporated.

There were two key areas where qualitative research and PPI were combined:

1. Study intervention vignettes, presented in further detail below, were co-developed in focused sessions for subsequent use in interviews and focus groups. This enabled the formal qualitative study to gain a diverse sample of participants’ perspectives of incentive interventions identified in systematic reviews and to assist with short-listing promising incentive strategies. By using vignettes, we aimed to include service user group members in the interpretation of systematic review findings (four evidence syntheses of the benefits of incentives for smoking cessation in pregnancy and breastfeeding, barriers and facilitators to maternal behaviour change and incentives for other lifestyle behaviours) [26, 39, 40] and to involve them as part of the research team.
2. A logic model was created from study findings to inform the design of incentive interventions as is recommended for public health interventions [41]. The logic model was presented to the groups towards the end of the study using interactive materials, which were adapted to suit the different formats of the groups. These included intervention components and processes for the delivery of incentive trials and how they fit with the findings from a narrative review of the qualitative evidence about the barriers and facilitators to behaviour change and maintenance for smoking cessation in pregnancy and for breastfeeding in women’s everyday lives [26]. These data contributed to the final version of the logic model and the assessment of its face validity.

In addition, members from both groups assisted with suggesting ‘harder-to-reach’ local candidates who might have different perspectives (e.g. teenagers, fathers) for recruitment to the main sample through affiliated NHS (National Health Service) staff and snowball sampling.

### Data analysis

Audio recordings of formal data collection sessions with the mother and baby groups were transcribed as soon as possible to facilitate accurate and comprehensive data documentation. Although the noise levels (children playing, shouting, etc.) compromised the digital recordings of discussions, especially with Group 2, GT also kept handwritten notes and transcribed the session recordings herself as soon as possible to facilitate accurate and comprehensive data documentation. These were circulated among the wider research team and entered into Nvivo10 software (QSR International, Burlington, MA) with other primary qualitative study data and analysed thematically following a Framework approach [42]. The qualitative research for this study is described in detail elsewhere [26].

Researcher reflexivity, where the researcher acknowledges and reflects on their effect(s) on the process and outcomes of research, is an important quality indicator in qualitative research and indeed any study. Our multi-disciplinary, multi-institution team and the selection of areas where health service attitudes to and experience of incentives were known to differ were *a priori* decisions to minimise bias of interpretation. Our research team included previous smokers, researchers with and without children, those with experiences of breast and formula milk feeding and male and female researchers. Researchers held different perspectives on incentive interventions for behaviour change, with four involved in incentive interventions (LB, DT, GT and PH). Differences and potential biases were discussed in regular team meetings. In addition, the researchers involved with the mother and baby groups (HM and GT) kept reflective diaries of their interactions and observations. Diary extracts were shared with the research team to facilitate consideration of influences that might inform group dynamics and involvement. Discussions occurred after every interaction and informed project development until study end in September 2013. An advance draft of this paper was also shared with a member of Group 1 for comment.

## Results

### Sample characteristics

Twenty-one women across the two groups were involved in various PPI activities on more than one occasion over a period of time. Nineteen of these women were also engaged more formally in qualitative research: nine in North East Scotland and ten in North West England. (See Appendix 1). Women were provided with clear information (verbal and written) about each stage of their involvement and asked to sign a consent form (consent was an ongoing process as women engaged in the different activities, including use of quotations in study publications) [34].

### Rapport-building, relationships with service users and unintended consequences

Both service user groups took evident pride in researchers coming into their territory. They were welcoming and hospitable, and offered refreshments and home bakes (Group 1) or computer facilities for directed sessions (Group 2). In addition, the researchers witnessed conversations that are seldom encountered in formal health or research settings. For example, some group members were adversarial towards health professionals; others described complex and disruptive domestic relationships, or expressed highly charged personal opinions towards health behaviours (smoking during pregnancy), which, on one occasion, resulted in someone leaving the room. In the presence of health service staff, such accounts would have been unlikely.

Stable membership meant there was a group culture/norm and that collaboration was through these terms in Group 1. This meant that members would not wish to challenge the group ‘norms’, although it did allow for informal conversations and the atmosphere was friendly and respectful. The Group 1 researcher (HM) was also invited to participate in social events. Of the six informal social events that took place during the course of this research, HM attended three to show goodwill (a Hallowe’en party, a Christmas fair and an end of term mums’ bingo night). Researcher participation can therefore be considered as ‘active’ [35] as HM was able to gain insight into the cultural codes and rules for behaviour of the group. The requirements of a researcher to remain objective and maintain a distance, compared with a more anthropological approach to fieldwork, were discussed with the wider research team. Informal contacts and balancing communication traffic presented a need for the researcher to be mindful of balancing PPI activities and remaining objective as in most formal qualitative research with pressure for full group membership.

The management and composition of Group 2 meant that these opportunities were not available so undertaking qualitative research was more challenging due to structural and organisational factors. Additional challenges were faced when trying to engage parents and carers in meaningful and uninterrupted conversations when their infants/children were present and noise levels (children playing, mothers talking, babies crying, etc.) compromised some audio recordings. The need to move around the room to approach members was a barrier to group engagement. The frequently changing group membership also resulted in more researcher time spent introducing these formal research processes than in Group 1. Children’s Centre staff supervised children when research activities involved concentration to read and discuss materials and computer facilities were made available to facilitate involvement of group members when needed. The drop-in format at Group 2 meant that the group was sufficiently anonymous to allow for people to express their thoughts and feelings; however, these could be too freely expressed and, on occasion, was identified to have caused offence. Flexibility was key and it was necessary to adapt rather than replicate methods according to group context and membership, which resulted in different experiences.

For both groups, long school holidays coincided with crucial research stages, for example the final qualitative data analysis overlapped with the school summer holidays.

Our collbaorations may have led to unintended consequences. Some group members appeared to change, or express desire to change, lifestyle behaviours during the involvement process, including stopping smoking and eating more healthily, while others did not. One group member joined a local health services committee as a lay member and enrolled on a further education college course.

### Relationships with wider research team

Whilst mother and baby group members were invited to attend research team meetings, and to meet with our advisory panel, they did not attend at either site. Therefore, engaging the mother and baby groups with the wider research team was problematic. The health visitor group facilitator for Group 1 attended one meeting (September 2012) and suggested that group members would have been very nervous, had any of them attended, because of the formality of the meeting. Her successor was invited to a subsequent meeting and expressed an interest, however, she did not attend. Travelling was an issue for members of Group 2; however, they did not accept an invitation to join meetings by telephone. Likewise, when research staff team members were invited to attend visits to the mother and baby groups, the idea was mostly rejected. Some made highly sceptical comments like *“you’ll be lucky!”* and *“ahem, no!* [laughing]*”* PH attended with HM at Group 1 on two occasions and NC attended with GT at Group 2 on one occasion, both to assist as observers of qualitative research activities.

### Substantive contributions from combining PPI and qualitative research

PPI members voiced some different perspectives from those found in the emerging main qualitative dataset. In the PPI activities, the researchers perceived that women were more candid when describing the barriers to changing their behaviour, whereas in the interviews, there seemed to be an element of ‘what the researcher might want to hear’. Members also assisted with recruitment of ‘harder-to-reach’ women through snowball sampling (i.e. by referral of friends and acquaintances) to ensure further inclusion of disconfirming data (new data that challenged what we had already found).

The substantive contributions made by combining and overlapping PPI and qualitative research are summarised below.

### Commenting on and interpreting the presentation of researcher constructed study vignettes derived from a systematic review prior to using them in qualitative interviews

#### Systematic reviews and study vignettes

The mother and baby groups contributed to interpreting systematic review findings by providing feedback on study vignettes derived from the evidence syntheses. Six vignettes were constructed by the research team from three smoking cessation and three breastfeeding studies. These studies were selected either because they had statistically significant effects or involved an unusual or innovative approach [43–48]. Different vignette structures were tried out, for example presenting and discussing an intervention chronologically and sequentially, revealing one bullet point at a time (the preferred option amongst the research team) compared to presenting the whole vignette. Various formats for the vignettes were also piloted (e.g. power point screenshot, written bullet points on one and/or multiple sheets, patient journey/map). Group discussions enabled us to identify that presenting the vignette as a whole and the power point screenshot were the most popular. Through discussion, this helped us to make study interventions more salient to facilitate assessment of the acceptability of components and processes, as well as to inform the development of a short-list of promising incentive strategies. This was particularly useful as very little detail around feasibility and acceptability of content and delivery processes was identified in the systematic reviews, even where included studies were classified as ‘qualitative’ or ‘mixed methods’ [26].

### Developing resources

#### ‘Ladder’ logic model

**Fig. 1:**
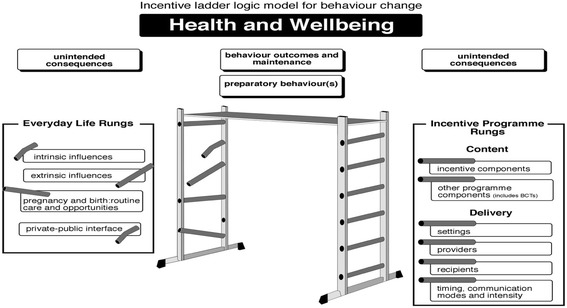
‘Ladder’ logic model

In the final stages of the study, a logic model that emerged from mixed methods analysis of the entire BIBS study was introduced to the groups (Fig. 1). The development of this model is described in the full report [26]. With mother and baby group involvement, our researcher-generated logic model for incentive intervention design was modified using the metaphor of a ladder to translate theory and findings into a lay tool which had face validity for potential trial participants.

**Fig. 2:**
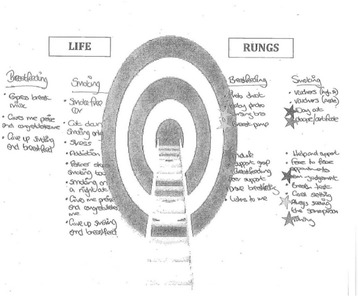
Group 1 – intervention ‘ladder’

As researchers, we wanted to garner a sense of how the logic model could be communicated in lay terms and also to assess whether it could be used with potential trial participants as a tool to contribute to identifying important trial components and processes to optimise intervention co-design. Two adaptations of the logic model were agreed through research team discussion to suit the different formats of the service user groups. For Group 1, it was presented as a blank ladder on a piece of A3 paper with three envelopes labelled ‘life’, ‘incentive’ and ‘other’ rungs (Fig. 2). Each envelope contained individual paper ‘rung’ cards with labels corresponding to the barriers and facilitators identified from a systematic review of the barriers and facilitators for stopping smoking and breastfeeding and from primary qualitative data [26], and some blank rungs for women to write their own contributions. Service users (*n* = 4) attempted to construct their own ‘ideal’ individually-tailored incentive interventions which they thought would facilitate the behaviour (smoking cessation and/or breastfeeding) in this interactive and practical session. They then prioritised intervention components and barriers/facilitators by applying star stickers to highlight those ‘rungs’ they considered crucial.

**Fig. 3:**
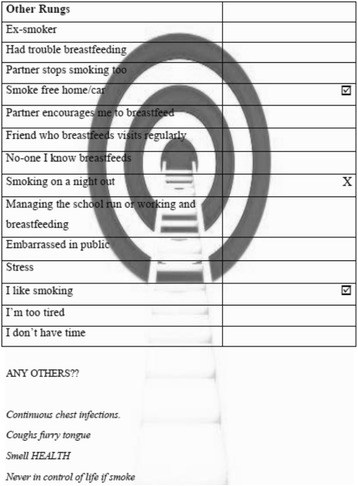
Group 2 – intervention ‘ladder’

In Group 2, due to children being present in the room, a different format was adopted. The researchers constructed separate A4 sheets, which had a ladder diagram picture in the background for the different ‘rungs’ (‘life’, ’incentive’ and ‘other’) (Fig. 3). Mothers (*n* = 4) engaged actively with the process and were requested to tick and/or star the rungs considered most important or relevant and a cross by those considered unimportant for behaviour change programmes.

**Fig. 4:**
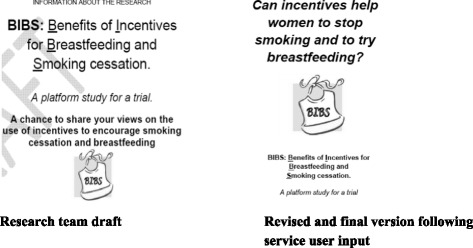
Comparison of the patient information leaflet before and following service user input

Service users engaged well with constructing ladders and this stimulated rich discussions about the choices they would make and the reasons why – what they would need and value within a trial –demonstrating face validity for the co-design of complex incentive interventions. Women found the ‘ladder’ idea easy to understand, relate to and use. More importantly, they found it acceptable as a model to consider how incentive behaviour change theory could be translated into their everyday lives. Some participants spontaneously talked about the implications for other health behaviours, in particular healthy eating, which they considered too expensive to do, suggesting the model may have wider relevance for addressing other health behaviours. However, others expressed entrenched resistance to change; for example, when completing a ladder for stopping smoking, one woman responded to breastfeeding saying “*no, no, no, no, no!”*

## Discussion

In our study, we combined PPI and qualitative research with some overlap and in Table 3 we summarise the key similarities and differences that we considered in doing this. We recognised that patient and public involvement (PPI) in all research is recommended and aimed to incorporate this in a meaningful way [22] from the earliest point and in as many stages as possible through early recruitment of mother and baby collaborator groups and ongoing interactions with them for the duration of the project [6]. We were also clear about ensuring that qualitative research was integrated within the mixed methods platform study design because it is crucial in the early stages of designing complex intervention trials [9]. What was less clear from the literature is how either/or can be optimised to ensure greater involvement of ‘harder-to-reach’ groups. This is especially important where the research is relevant for specific populations’ benefits and where they may be opportunities in combining both. Because approaches and reporting standards vary for PPI [20], but tend to be more rigorous in qualitative research [13, 14], the latter approach appears to be more pronounced. In our study, we were able to combine both. We involved community groups [17] representative of the target population to include their perspectives on the research by involving them in PPI activities. We also developed partnerships with the groups to facilitate qualitative research with their members. We described both clearly in our final report [26].

**Table 3:** Summary of key similarities and differences between PPI and qualitative research

	Similarities	Differences
Why	Both PPI and qualitative research aim to incorporate deeper understanding of the research problem and ensure greater relevance of the findings to society. Both were used to gather information to help in the design of an intervention and potential clinical trial.	PPI involves non-researchers and non-clinicians in research to inform study design and conduct. Qualitative research involves collecting data from participants to answer the research question(s).
Who	Both PPI and qualitative research can include representatives of the target population of the study.	PPI might only include representatives of patients or the public in general rather than the target population and representatives might be trained in PPI. PPI representatives are usually fewer in number than researchers or research participants. Qualitative research might seek to include broader perspectives and disconfirming data from as diverse a sample as possible.
What	Both PPI and qualitative research (with consent) can collect data using traditional methods such as recorded discussions, interactive sessions, and activities. Any collection of data for research purposes, audio-recording or subsequent use of quotations requires research ethics committee approval (http://www.hra.nhs.uk/).	PPI is predominantly involvement in the tasks of research and is a two way exchange of knowledge that influences study design, whereas qualitative research is predominantly for advancing understanding and thus involves the researchers being informed by the participants. Qualitative research requires research ethics committee approval whereas PPI usually does not.
Where	Both PPI and qualitative research can take place in a range of settings, including Universities or public spaces and either face-to-face, by telephone or using remote audio-visual technology.	PPI tends to involve inviting representatives to join research team meetings in academic settings, but can include researchers going out into the community. The setting for qualitative research takes into account participant preferences and where is best for the data collection.
When	Both PPI and qualitative research can involve single or serial interactions or meetings.	PPI is more likely to take place over an extended period and involve multiple meetings. Qualitative research is more likely to involve a one-time data collection session.
How	Both PPI and qualitative research might employ similar purposive sampling approaches to represent specific populations.	PPI is more likely to draw on established networks of people interested in contributing to research. Qualitative research designs vary based on the aims of the study, e.g. snowball, stratified, theoretical, purposive and convenience sampling [42].

Our approach shows that physically going to and engaging with PPI in ‘harder-to-reach’ group settings [19] – relevant for health behaviours where there are strong social gradients – is valuable. Going to the groups, on their terms, flexibly and gradually enabled us to build trusting rapports and to see issues through the participants’ eyes. This participatory approach also enabled less ‘professionalised’ PPI [38]. This contrasts with trained PPI where there is potential for representatives to be influenced more by the academic community (e.g. by receiving training to understand research language so that they can contribute in research team meetings where academics outnumber PPI). In our study, PPI representatives outnumbered the researchers.

Combining PPI and qualitative research can add value to studies informing the design and delivery of complex interventions which target ‘harder-to-reach’ populations. This is because PPI can contribute additional information to research data when combined with formal data collection, for example if perspectives are being voiced that are different to those being heard through traditional qualitative data collection methods [30], as happened in our study. Qualitative interviewing is primarily a guided conversation where knowledge, views and experiences are provided by the participants in response to open questions and prompts by the interviewer. Often great care is taken in how the researcher contributes, with the research team carefully constructing an introduction and topic guide in order to avoid leading or priming a participant’s response. The main difference with PPI is that there is more two-way sharing of knowledge, views and experiences within a research team and this tends to be around the ‘doing of’ research. Power differentials are played out in different ways in that PPI can contribute more to informing what and how research is done. Our approach resulted in new perspectives from the target populations for potential smoking cessation and breastfeeding incentive intervention trials, which might not have been gained with more traditional methods of engagement with PPI or qualitative research alone. PPI can advise and help to refine research processes so that they assist in recruiting ‘harder-to-reach’ groups or translate research findings in a way that is meaningful for these groups [22]. Their suggestions modified our approach, our language, our strategies to facilitate involvement of further relevant perspectives and translation of a logic model to a lay audience. Working with two groups in different areas taught us that there is no set formula or protocol to engaging service users through their groups and involvement benefits from being adapted to the particular context. In particular, seating arrangements, the structure of the room and drop in formats were not suitable for focused group activities.

However, in our study, the distinction between PPI and qualitative research was subtle as there was overlap and movement between PPI and qualitative research throughout. In practice, groups or individuals had multiple roles in that they were influencing decisions, making substantive contributions to research processes, generating data and providing feedback on the results. This created a need to negotiate boundaries between the researchers and the researched where the lines between objective observation and subjective participation required careful consideration.

Ethical approval was gained, although it is not required for PPI, and our study meets the principles of good PPI [31, 32]. We considered ethics committee approval necessary to meet international guidance [33] to obtain informed consent to audio record discussions to contribute to qualitative research data; use research team generated topic guides; use interactive session outputs in study publications and more generally for documenting, managing and reporting on both PPI and qualitative data [26]. We consider that ethical approval for these types of activities is something that needs to be addressed in future guidelines for best practice in PPI. In addition, it was important to consider issues of burden and ability to opt out or remove data at any stage, particularly so individual members did not feel under any obligation given that fees were negotiated in advance for group involvement and that potential identification was possible. The groups were given the option of anonymity but chose to have their involvement acknowledged by group name in study publications. This raises issues for those considering similar models of PPI where anonymity may be more difficult to preserve.

Co-applicant PPI collaboration is a strength for any complex or contentious research, especially where the target population(s) is ‘harder-to-reach’. The use of diverse, iterative and flexible approaches allows adaptation and integration of PPI and qualitative research methods. This strengthened our results because new findings were included and these were accessed via the relationships with women in the groups rather than their familiarity with being involved in research. In addition, they were not only ‘research participants’, but were also helping us to frame our research study and to explore insights from other studies, which enabled us to have more open and frank conversations. Working with groups over an extended period of time throughout the research project built trust, which meant that our research went beyond either involvement or qualitative research. Another benefit of our approach was the sense of pride expressed by group members about being involved in the research and hosting researcher visits, which challenged traditional power relations.

A key strength of our approach is to combine the complementary features of PPI with qualitative research, so that they enrich the contributions that each make to the research questions and the study design. Flexible, sensitive and reflexive approaches were needed to achieve rich engagement from what are often considered ‘harder-to-reach’ groups. This is one of few primary research accounts of the issues encountered when doing this, in particular our consideration of ethical issues. Potential limitations of combining both are the blurred boundaries between PPI and qualitative research, and the challenges of pre-emptively describing the participatory approach in order to obtain ethical approval. From the perspectives of the women involved, they became more familiar with the research as their PPI matured. By incorporating informed consent and opt-out options throughout, over-burdening of representatives was prevented. Our separate qualitative sample ensured that we incorporated a broader range of views, and the mixed methods involved in the wider project allowed us to include the perspectives of a number of populations (e.g. the MORI survey of the general public [49]).

A limitation of combining PPI and qualitative research is the possibility of ‘group think’ [26].

We sought to minimise this, however: first by working with two groups and second by using other strategies within the research [26]. Although group members contributed to the development work leading to the shortlist of incentive strategies asked about in the MORI survey of the general public [49], a separate sample of independent general public participants was sought to pilot this to minimise the ‘group think’ that may occur through repeated discussion of a topic. Our approach involved considerable researcher time. This will not add value to every project. Therefore, the benefits need to be weighed against resources available. The debate around how far to take PPI without compromising research outcomes is also pertinent [22]. For example, PPI collaborators may not consider reflexivity and bias in the same way/s as trained researchers, which may limit the role they have and stages in which it is appropriate for them to be involved, and may influence the qualitative data that they contribute, depending on when it is collected in relation to the PPI. This is especially relevant for ensuring that ‘lay’ voices remain ‘lay’ and are not obscured by research training or over-professionalisation of involvement by experience or desensitisation. Undertaking qualitative research that overlaps with PPI requires re-definition of roles and relationships in this regard and reflexivity in reporting. In addition, the extent to which the collaboration might have primed individual motivation to behave differently is uncertain and could be a limitation. However, despite some exceptions, many PPI participants remained highly resistant to change in relation to either stopping smoking and/or breastfeeding. Involvement with the wider research team was usually dependent on single researcher observations rather than PPI within a wider team (e.g. at project meetings). This often applies to qualitative research data collection, however, this is a potential limitation to consider when comparing PPI with qualitative research.

We recommend that each research team for every study considers whether and how both PPI and qualitative research might contribute to or compromise research outcomes and if there are opportunities to combine the two for maximum benefit to all stakeholders. How each can contribute is likely to depend on the research question being addressed, the stage of the research and the design of the study. When ‘harder-to-reach’ service user perspectives are crucial to the research, funding bodies should consider how early involvement in the design and application stages of a grant can be achieved. However, for all PPI in future, greater attention needs to be paid to maximising opportunities for researchers to work more flexibly with the most relevant PPI representatives, who might not always be those who are trained for involvement in research. Consideration should also be given to PPI representatives’ social context(s) to ensure that these can be approached in reflexive and sensitive ways that support involvement and mutual benefits for the research and those who contribute PPI.

## Conclusions

Combining and overlapping PPI and qualitative research added ‘harder-to-reach’ contributions from members of the target populations to trial design research beyond what could be achieved through PPI or qualitative research alone. Extensive involvement of ‘harder-to-reach’ service user groups as collaborators across a range of research activities in their own research settings rather than in an academic setting enabled less ‘professionalised’ PPI that benefited the progression of the study. Collection of formal qualitative data from PPI service users added disconfirming data.

There are, however, implications of our approach for other research teams. These include further consideration of: who, where and how to engage PPI representatives in research; the interface between PPI and qualitative research and their underpinning logic models; how researchers work flexibly with people and understand the context in reflexive and sensitive ways; the scope and remit of ethical approvals and the pros and cons of providing training for PPI representatives.

## Appendix

### Appendix 1

**Table 4:** Table of participants in qualitative research

	Parent status^a^	Age	Marital Status^b^	Ethnicity^c^	Education^d^	Employed (yes/no)	Smoking Status^e^	Lives with smoker (yes/no)	Infant feeding intentions or feeding status^f^	Previous Infant Feeding Experiences^g^	Experience of Incentives^h^
North East Scotland	Mother	21	1	1	3	No	5	Yes	3	2	No
	Mother	40	1	1	3	Yes	1	No	2	1	No
	Mother*	30	1	1	2	No	1	No	1	1	No
	Mother*	22	2	1	3	Yes	1	No	3	1	No
	Mother	32	1	1	3	No	5	No	N/A	1	No
	Mother	Unknown	1	1	Unknown	No	5	Yes	3	1	No
	Mother*	Unknown	2	1	Unknown	No	5	No	3	1	No
	Grandmother	51	1	1	3	Unknown	5	Yes	N/A	Unknown	No
	Mother	Unknown	2	1	Unknown	No	3	No	3	1	No
North West England	Mother	62	1	1	3	No	1	No	N/A	1	No
	Mother*	26	1	1	3	Yes	1	No	1	1	No
	Mother	28	1	1	3	Yes	1	No	N/A	1	No
	Mother*	27	1	1	3	Yes	4	No	1	1	No
	Mother	39	2	1	3	No	1	No	N/A	1	No
	Mother	53	2	1	1	No	1	No	N/A	1	No

NB demographic details are available for all those who took part in audio recorded qualitative research sessions (*n* = 15 - 9 from Scotland and 6 from England)

^a^Mother relates to those who have older children (who may/may not be currently pregnant); mother* relates to women who have older children and who are pregnant; Woman relates to those who are pregnant/expecting first child

^b^1 – participant married/living together/in a relationship; 2 – single/divorced

^c^1 - White; 2 - Black or Minority Ethnic classifications (BME)

^d^1- Degree level qualification; 2 - A level or equivalent; 3 - GCSE/NVQ or equivalent; 4 - No formal qualifications; 5- not recorded

^e^1 – Never smoked; 2 – Quit during pregnancy; 3 – Cut down during pregnancy; 4 – Quit prior to pregnancy; 5 – Currently smoking

^f^Code relates to women who are currently pregnant or have a baby under 6 months; 1 – Plan to breastfeed/breastfeeding; 2 – Plan to mixed feed/mixed feeding; 3 – Plan to formula feed/formula feeding

^g^.Code relates to families with older children/interviewed in post-natal period 1 – Previous experience of breastfeeding; 2 – Never breastfed

^h^‘Other’ relates to those involved in Barnardo’s Early Years Early Action Fund; http://www.barnardos.org.uk/media_centre/press_releases.htm?ref=81644)

### Appendix 2

**Table 5:** Standard PPI

Designing and editing study materials	Protocol and study information materials	When the study started (February 2012) and before we received ethical approval, service users assisted in developing the protocol and study information materials through third party feedback to their group’s representatives. Most notably, they helped us to rephrase several sections of the information sheet for both readability and acceptability (Fig. 4). Revisions took account of target population participants’ style preferences, language and social and cultural contexts. In particular, they drew our attention to their unfamiliarity with the term ‘cessation’ and the predominance of formula feeding, either from their own personal experience or in their immediate family and social networks. ‘Help women to stop smoking’ and ‘try breastfeeding’ were more appropriate phrases because women pointed out that ‘smoking cessation’ is too technical, and ‘encourage’ is persuasive, while the word ‘breastfeeding’ alone implies an assumption or certainty. The last of these was a very important change as we noted that a mother in one of the groups disclosed that she was providing exclusive breast milk to her infant on her characteristics form. However, at meetings, she allowed others group members to believe that she was formula feeding, using bottles of expressed breast milk, but talking about preparing formula feeds. As breastfeeding was not a social norm in the group, perhaps she anticipated that it could be considered unacceptable by some and preferred to ‘hide’ her choice to ensure her involvement and acceptance within the group.
Piloting study tools	Interview schedules	We piloted draft interview topic guides in three focus groups with service user mother and baby groups (Groups 1& 2) and with individual women (Group 2) prior to recruiting participants to the formal qualitative research. In Group 1, this involved trying a structured topic guide, the integration of study vignettes (described below) within the schedule and language/format revisions. The final, preferred version was unstructured with prompts for use if and when appropriate. For example, opening with questions around what incentives were/meant for women – so using women’s conceptions of incentives to guide the interview.
	DCE	This was piloted with four mothers with a history of smoking in Group 2 using online simulation. When reading and answering each of the questions (using *Survey Monkey*’s online format (www.surveymonkey.com)), the mothers were asked to use the ‘think aloud’ cognitive interviewing technique [50] whereby they expressed their feelings and discussed any issues around the questions/process. This session was facilitated by the researcher (GT) who audio recorded and transcribed key points for team discussion. All participants in this session took the questionnaire seriously and engaged with the choices. Descriptions and explanations in the DCE were revised for better understanding and readability based on their comments. There were pros and cons of piloting the survey with an established group, particularly when a more diverse sample, representative of the general public, is sought. However, in this instance, all women were current or previous smokers (the sample population who would complete the DCE), and so it was important to pilot with these members of the group.
Study outputs	Lay summary	Mothers in Group 1 read the first draft of the lay summary and commented. Two sentences were reworded according to their feedback and ‘promising’ was replaced with ‘potential’ as it was felt that the meaning of the former could not be easily understood without explanation and examples.
	Dissemination	We took a poster presentation [39] to discuss with Group 1 in October 2012 to show them how we are reporting and presenting results publicly. They liked the format, which was something none of the members had seen before, and were delighted that their group’s name had been included, expressing a sense of immense pride regarding their involvement. At the final group meetings (Groups 1 & 2), mothers offered numerous suggestions for dissemination, such as face-to-face briefings, making information available online on key web pages that parents may access, leaflets within community/health facilities, inclusion within school newsletters and local newspaper articles with a group and researcher photograph. Several members requested personal copies of the published final report. A member of Group 1 has read and commented on the final draft of this paper. She raised the point that papers such as this are aimed at academic and professional audiences and she has expressed an interest in working with us to communicate our findings to others

**Table 6:** Qualitative research

Observations	Researchers observed the groups on a number of occasions. HM attended Group 1 during eleven separate visits and participated in three social events, using an ethnographic approach. PH observed at two visits. GT visited Group 2 four times. NC observed and assisted GT at one meeting. Reflexive diaries were kept by all researchers with notes being shared within the research team.
Interviews	Formal individual interviews were undertaken with two members of Group 2 using the study participant information sheet and consent forms, following the study protocol. [34]
Focus groups	Formal focus groups were undertaken twice with members of Group 1 (*n* = 5; *n* = 5) and once with members of Group 2 (*n* = 4) using the study participant information sheet and consent forms, following the study protocol. [34]

In addition, qualitative interviews for this study were undertaken with 88 pregnant women/recent mothers/partners, 53 service providers, 24 experts/decision-makers and 63 conference attendees [26]

### Appendix 3

ᅟ

## Additional file

Additional file 1:
**Socio-demographic, smoking and breastfeeding experience characteristics form.** (PDF 187 kb)
