# Supplementary material for: Combining PPI with qualitative research to engage ‘harder-to-reach’ populations: service user groups as co-applicants on a platform study for a trial
Source: Res Involv Engagem. 2016 Mar 24;2:7. doi: 10.1186/s40900-016-0023-1 (PMC5611582; doi:10.1186/s40900-016-0023-1)
Supplement: Additional file 1: — Socio-demographic, smoking and breastfeeding experience characteristics form. (PDF 187 kb) [file 40900_2016_23_MOESM1_ESM.pdf]

To be completed by researcher:

Researcher	Area	Mother's Code

BIBS: Benefits of Incentives for Breastfeeding and Smoking cessation

Pregnant women/new mothers

Please can you complete the following details about yourself:

***Please delete as appropriate**

1) **Your age:** ☐☐

2) **The first four digits of your postcode:** ☐☐☐☐

3) **Please record your relationship status by ticking the appropriate box:**

Marital Status	*Please tick the box that applies*
Married	
Living together	
In relationship (but not living together)	
Single	
Widowed	
Divorced/separated	
Civil partnership	

4) **Please record your ethnic background by ticking the appropriate box:**

Ethnic Background		*Please tick the box that applies*
White	White British	
	Irish	
	Other white background	
Mixed Race	White and black Caribbean	
	White and black African	
	White and Asian	
	Other mixed background	
Asian/Asian British	Indian	
	Pakistani	
	Bangladeshi	
	Other Asian background	

Black or Black British	Caribbean	
	African	
	Other Black background	
Chinese or Other Ethnic Groups	Chinese	
	Other ethnic group	

5) **Please record your highest qualification by ticking the appropriate box:**

Qualification	*Please tick the box that applies*
O Grade, Standard Grade, Access 3 Cluster, Intermediate 1 or 2, GCSE, CSE, Senior Certificate or equivalent	
SCE Higher Grade, Higher, Advanced Higher, CSYS, A Level, AS Level, Advanced Senior Certificate or equivalent	
GSVQ Foundation or Intermediate, SVQ level 1 or 2, SCOTVEC Module, City and Guilds Craft or equivalent	
GSVQ Advanced, SVQ level 3, ONC, OND, SCOTVEC National Diploma, City and Guilds Advanced Craft or equivalent	
HNC, HND, SVQ level 4 or equivalent	
Degree, Postgraduate qualifications, Masters, PhD, SVQ level 5 or equivalent	
Professional qualifications (for example, teaching, nursing, accountancy)	
Other school qualifications not already mentioned (including foreign qualifications)	
Other post-school but pre-Higher Education qualifications not already mentioned (including foreign qualifications)	
Other Higher Education qualifications not already mentioned (including foreign qualifications)	
No qualifications	

6) **What is your current occupation/job:**

____

____

7) **When is your baby due (or if your baby has been born)? Please provide the month and year**

M	M	Y	Y

8) **Where do you intend to/did you give birth* (we would like this information in order to know under which NHS organisation your maternity care is registered)?**

____

9) **How many ‘other’ (e.g. older) children do you have?** ☐

10) **Smoking history:**

Please answer the following questions by ticking the appropriate boxes

a) Have you ever smoked cigarettes?

Yes *(if yes, please go to question b)* ☐

No *(if no, please go to question g)* ☐

b) Have you smoked at all in the last two years?

Yes *(if yes, please go to question c)* ☐

No (*if no, please go to question e)* ☐

c) Do you smoke cigarettes at all now?

Yes *(if yes, please go to question d)* ☐

No *(if no, please go to question e)* ☐

d) Did you/have you smoked cigarettes at all during your most recent pregnancy, after you found out you were pregnant?

Yes *(if yes, please go to question f)* ☐

No *(if no, please go to question g)* ☐

e) When did you finally give up? *Please tick one box only (and then go to question g)*

Before you knew you were pregnant ☐

As soon as you found out you were pregnant ☐

Later on during your pregnancy ☐

After the birth ☐

f) Since you knew about your most recent pregnancy, did you do any of the following DURING your pregnancy?
Please tick one or more boxes (then go to question g)

Cut down ☐

Gave up but started again ☐

Increased the amount you smoked ☐

None of the above ☐

g) During your most recent pregnancy, did any of the people you lived with smoke cigarettes?
Please tick one or more boxes (then go to question h)

Yes, my partner smoked ☐

Yes, someone else I lived with smoked ☐

No, nobody else who I lived with smoked ☐

Not applicable – I lived alone ☐

h) Do any of the people who live with you now smoke cigarettes?
Please tick one or more boxes

Yes, my partner smokes ☐

Yes, someone else I live with smokes ☐

No, nobody else who I live with smokes ☐

Not applicable – I live alone with my baby ☐

11) Feeding history

If you are pregnant at the moment please go to question a) – if you have recently had a baby, please go to question b)

a) If you are pregnant now, how do you plan to feed him/her? (*Please tick one of the boxes and then go to question e)*

Infant formula feed ☐

Breastfeed ☐

Breastfeed and infant formula feed ☐

Have not decided ☐

b) If you have recently had a baby - thinking about the milk that your baby has been given over the last 7 days has he/she had… *(please tick one of the boxes and then go to question c)*

Only breast milk ☐

Only infant formula milk ☐

Breast milk and infant formula milk ☐

c) Has your recent baby EVER been given infant formula milk, even if this was only once? *(please tick one of the boxes and then go to question d)*

Yes (even if only once) ☐

No ☐

d) Has your recent baby EVER been given breast milk or have you put your baby to the breast, even if this was only once? *(please tick one of the boxes and then go to question e)*

Yes (even if only once) ☐

No ☐

e) If you have any older children – did you breastfeed any of your older children?

Yes / No / Not Applicable*

If you answered yes – please can you record the longest amount of time any of your children were breastfed for? (please indicate how many days, weeks, months or years as appropriate in the box below):

Days	Weeks	Months	Years

12) **Experience of incentives**

Have you ever been part of an 'incentive' scheme which provides you with gifts or services to help you to change any lifestyle behaviour?

Yes / No*

If you answered yes please detail below which lifestyle behaviour the incentive scheme was targeting?

____

Please can you tell the researcher more about the incentive scheme during the discussion

THANK YOU FOR COMPLETING THIS FORM
